# Association between glycemic variability and short-term mortality in patients with acute kidney injury: a retrospective cohort study of the MIMIC-IV database

**DOI:** 10.1038/s41598-024-56564-7

**Published:** 2024-03-11

**Authors:** Yifan Guo, Yue Qiu, Taiqi Xue, Yi Zhou, Pu Yan, Shiyi Liu, Shiwei Liu, Wenjing Zhao, Ning Zhang

**Affiliations:** 1 Department of Endocrinology and Nephropathy, Wangjing Hospital of China Academy of Chinese Medical Sciences, Beijing, China; 2 Graduate School, Beijing University of Chinese Medicine, Beijing, China; 3 Department of Endocrinology, Miyun Hospital District, The Third Affiliated Hospital of Beijing University of Chinese Medicine, Beijing, China; 4 Department of Nephropathy, Xiyuan Hospital of China Academy of Chinese Medical Sciences, Beijing, China; 5 Department of Nephrology, Beijing Hospital of Traditional Chinese Medicine, Capital Medical University, Beijing, China

**Keywords:** Endocrinology, Nephrology, Risk factors

## Abstract

Acute kidney injury (AKI) represents a significant challenge to global public health problem and is associated with poor outcomes. There is still considerable debate about the effect of mean blood glucose (MBG) and coefficient of variation (CV) of blood glucose on the short-term mortality of AKI patients. This retrospective cohort study aimed to explore the association between glycemic variability and short-term mortality in patients with AKI. Data from the Medical Information Mart for Intensive Care IV (MIMIC-IV) database were analyzed, including 6,777 adult AKI patients. MBG and CV on the first day of ICU admission were calculated to represent the overall glycemic status and variability during the ICU stay in AKI patients. The primary outcome indicator was ICU 30-day mortality of AKI patients. Multivariate Cox regression analysis and smoothed curve fitting were used to assess the relationship between blood glucose levels and mortality. Eventually, the ICU 30-day mortality rate of AKI patients was 23.5%. The increased MBG and CV were significantly correlated with ICU 30-day mortality (hazards ratio (HR) = 1.20, 95% confidence interval (CI) 1.14–1.27; HR = 1.08, 95% CI 1.03–1.13). The smoothed curve fitting showed a U-shaped relationship between MBG on the first day of ICU admission and ICU 30-day mortality (inflection point = 111.3 mg/dl), while CV had a linear relationship with 30-day ICU mortality. Thus, we conclude that MBG and CV were significantly associated with short-term mortality in intensive care patients with AKI. Tighter glycemic control may be an effective measure to improve the prognosis of patients with AKI.

## Introduction

Acute kidney injury (AKI) is characterized by a syndrome of rapid decline in renal function over a short time caused by various etiologies. It is typically manifested by a decrease in glomerular filtration rate (GFR) and a rapid increase in serum creatinine (Scr), with or without a decrease in urine output^1^. It has been reported that the incidence rate of AKI in intensive care units (ICUs) has exceeded 50%, imposing a significant burden on global healthcare costs^2,3^. In recent years, significant progress have been made in the diagnosis and treatment of AKI, but it is worth acknowledging that AKI remains a serious global public health problem^4^.

Blood glucose (BG) levels are an important indicator reflecting the status of glucose metabolism in the organism. In the human body, a delicate mechanism exists to regulate the dynamic balance of BG source and destination, ensuring the relative constancy of BG concentration. This intricate process is under the joint regulation of the nervous system, hormones, and various tissues and organs^5,6^. Previous studies have shown that factors such as infection, surgery, trauma, certain diseases, and medications have been the major causes of stress-related glycemic elevations in the ICU. Moreover, hyperglycemia has been identified as a significant influence of poor outcomes among critically ill patients^7,8^. A study has revealed that AKI patients face an elevated risk of experiencing both hyperglycemia and hypoglycemia. Consequently, adopting a broader BG concentration target, such as < 180 mg/dl or 140–180 mg/dl, may be more suitable for ICU patients with AKI compared to the previously recommended target range of 80–110 mg/dl^9^. Another study indicated that BG in AKI patients should be maintained at the reasonable levels (≥ 5.52 mmol/L), but the optimal control levels that suitable for AKI patients are not known^10^. Hence, whether more stringent glycemic control is needed in the ICU remains a controversial topic.

Mean blood glucose (MBG) and coefficient of variation (CV) have become significant indicators of glycemic control levels^11^. Compared with single BG, the MBG offers a better reflection for the general status of BG, while the CV provides a clearer depiction of the degree of BG fluctuations^12,13^. Studies have shown that both early hypoglycemia and increased glycemic variability are independent predictors of mortality risk in critically ill patients^14–16^. In patients with sepsis, Lu et al. found that overall glycemic variability during the ICU stay seemed to be more associated with disease prognosis than early BG fluctuation^12^. However, few studies have focused on the optimal goals of BG control in AKI patients. Additionally, the impact of glycemic variability on outcomes in AKI patients remains poorly understood. Consequently, further investigations are warranted to elucidate these aspects. Thus, we performed a retrospective study to explore the association between MBG and CV levels during the first day of ICU admission and 30-day mortality rates in AKI patients.

## Methods

### Data source

This retrospective cohort study was carried out in accordance with the Strengthening the Reporting of Observational Studies in Epidemiology (STROBE) guidelines^17^ and the tenets of the Declaration of Helsinki. We included AKI patients from the Medical Information Mart for Intensive Care-IV (MIMIC-IV v2.1) database (https://mimic.mit.edu). This is a single-center, longitudinal database that contains the medical records of 523,740 in-patients who received care at the Beth Israel Deaconess Medical Center from 2008 to 2019^18^. The database can be linked to the social security database to obtain information concerning out-of-hospital deaths^19^. Users can access the length of stay, demographic characteristics, vital signs, laboratory tests, medication usage and other information of each patient using a unique code assigned during admission. To protect the patient’s privacy, all personal information was de-identified, and only a random code was used to identify specific patient. Therefore, the requirement for informed consent and ethical approval was waived by the Ethics Committee of Wangjing Hospital of China Academy of Chinese Medical Sciences. An author of our current study had completed the Collaborative Institutional Training Initiative (CITI) course and passed both the “Conflicts of Interest” and “Data or Specimens Only Research” examinations (Certification ID: 9018458) and was thus authorized to download the MIMIC-IV database from the PhysioNet online forum (https://physionet.org/). Some or all code that support the findings of this study are available from the corresponding author upon reasonable request.

### Participant selection

**Figure 1:**
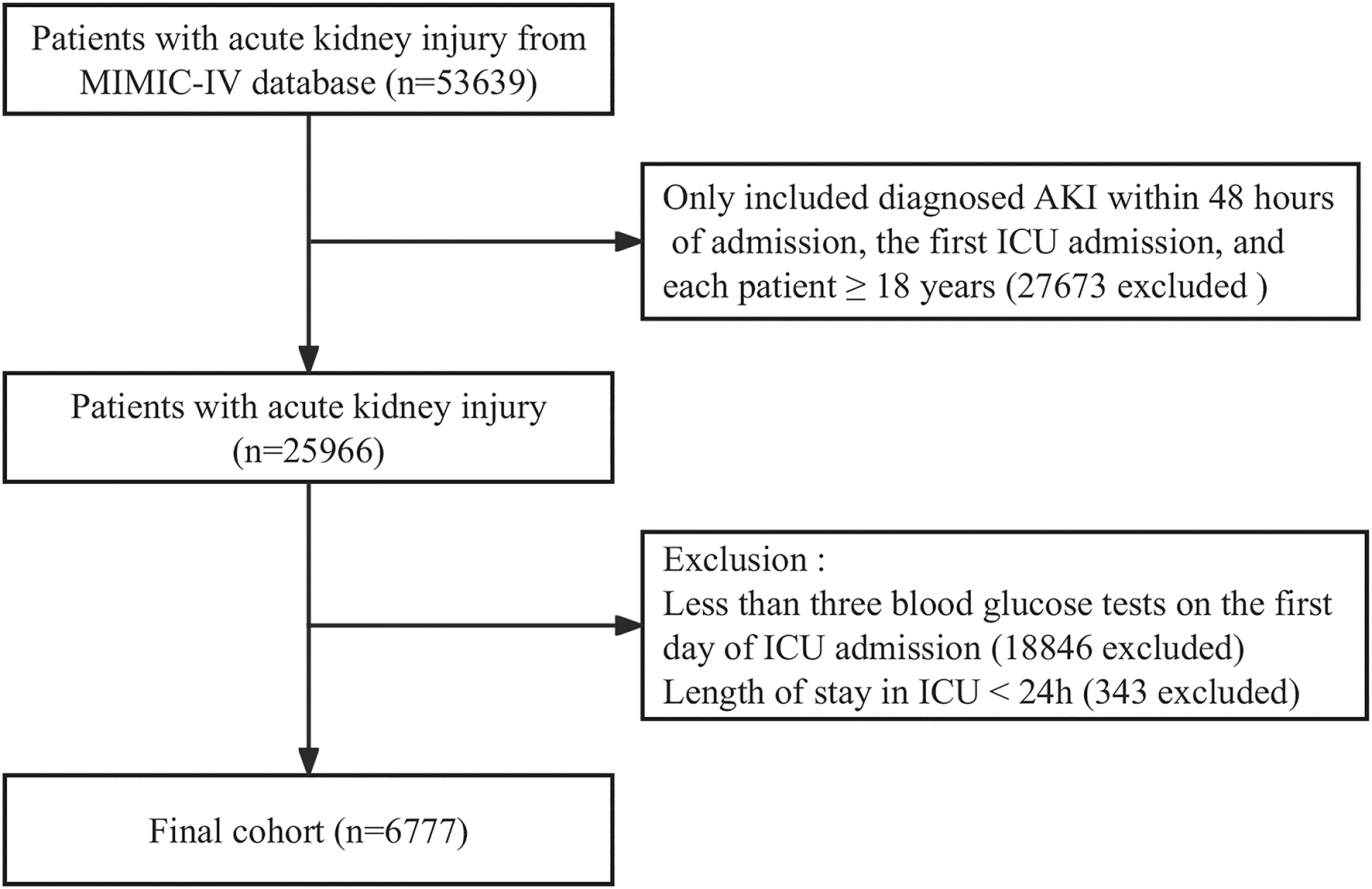
Flowchart of participant selection.

A total of 76,540 admissions were admitted to the ICU in the MIMIC-IV database, of which 53,150 were admitted to the ICU for the first time. According to the Kidney Disease: Improving Global Outcomes (KDIGO) guidelines^20^, AKI was diagnosed as follows: serum creatinine ≥ 1.5 times baseline or increase of ≥ 0.3 mg/dL within any 48 h period, or urine volume < 0.5 mL/(kg·h) for ≥ 6 h. All participants were required to meet the following inclusion criteria: (a) diagnosed AKI within 48 h of admission; (b) patients admitted to the ICU for the first time; and (c) aged ≥ 18 years. Exclusion criteria included: (a) BG records less than 3 times during the first day of ICU admission; (b) stayed < 24 h in the ICU. Ultimately, 6777 patients with AKI were included in the study (Fig. 1).

### BG measures and glycemic variability definition

We calculated the mean blood glucose (MBG) of the subjects using all biochemical glucose values on the first day of ICU admission. Moreover, to evaluate glycemic variability, we computed the coefficient of variation (CV) for glucose levels. The CV is calculated as the ratio of the standard deviation (SD) of BG to the MBG. We classified the MBG and CV into three categories according to the tertiles levels (T1 < 33.3th, T2 33.3–66.7th, T3 > 66.7th). Here, we used the first category of MBG and CV as reference values for comparison in our analysis.

### Variables

Baseline variables for the first 24 h of ICU admission were gathered from the MIMIC-IV database. This included demographic variables such as age and sex. Vital signs comprised heart rate, systolic blood pressure (SBP), diastolic blood pressure (DBP), and percutaneous oxygen saturation (SpO2). Laboratory tests covered hemoglobin, platelet count, white blood cell (WBC) count, sodium (Na), creatinine (Cr), and blood urea nitrogen^21^. Comorbidities recorded encompassed myocardial infarct, congestive heart failure, peripheral vascular disease, cerebrovascular disease, liver disease, kidney disease, malignant cancer, sepsis, diabetes, and hypertension. Treatment interventions catalogued included renal replacement therapy (RRT), mechanical ventilation, vasoactive drugs, and hypoglycemic drugs. Additional variables documented were the stage of AKI, sequential organ failure assessment (SOFA) score, and comorbidity index. The data were extracted by running Structured Query Language (SQL) using PostgreSQL software (v13.9.3) and Navicate Premium software (v16.0.6).

### Outcome

The study primary outcome was ICU 30-day mortality, defined as survival status at 30 days of ICU admission. The follow-up period was from ICU admission to ICU discharge or death. In addition, in-hospital mortality and the incidence of hypoglycemia were cited as secondary outcomes.

### Statistical analysis

All continuous variables were presented either as the mean ± SD for normally distribution data or median interquartile range (IQR) for non-normally distribution data. Comparisons across groups were conducted using *one-way ANOVA* or the *Kruskal‒Wallis rank-sum test.* Categorical variables were described as frequencies (%) and comparisons were made using the chi-square test (χ^2^). Multiple imputations were applied for missing data^22^.

We used univariate analysis to explore the preliminary relationship of all variables with ICU 30-day mortality. Multivariate Cox regression analysis was performed separately to determine the association between MBG, CV, and ICU 30-day mortality of AKI patients. In determining confounders, we took into account both clinical significance and insights from previously published relevant papers. We used covariate screens or identified as confounders those variables that changed the effect estimate by ≥ 10%. MBG and CV were analyzed as both continuous and categorical variables, and potential confounders were adjusted gradually in the four models. Model 1 was adjusted only for age and sex. Model 2 included further adjustments for heart rate, SBP, DBP, SpO2, platelets, WBC, Cr, and BUN. Model 3 expanded upon this by also adjusting for myocardial infarct, cerebrovascular disease, liver disease, kidney disease, malignant cancer, sepsis, hypertension, and diabetes. Model 4 incorporated additional adjustments for AKI stage, CRRT use, mechanical ventilation, vasoactive drug use, hypoglycemic drug use, SOFA score, and comorbidity index.

We used restricted cubic spline models to examine the possible nonlinear association between MBG, CV, and the incidence of ICU 30-day mortality. Additionally, we performed an inflection point analysis to identify any nonlinear relationships. ICU 30-day survival curves were plotted by Kaplan–Meier survival curves according to the tertiles of MBG and CV. These curves were then analyzed using the log-rank test. To further analyze the effects of general information, comorbidities and major interventions on the study outcomes, subgroup analyses were performed using a hierarchical Cox regression model. Subgroups were defined based on age, sex, comorbidities (including kidney disease and diabetes), interventions (including RRT and hypoglycemic drug use) and AKI stage. These analyses aimed to assess the reliability and robustness of the findings. At the same time, interactions between subgroups were examined using a variance ratio test. To ensure the robustness of our findings, we performed sensitivity analysis by Cox regression models for the data before interpolation and after removing all the missing data.

All analyses were performed using the statistical software packages R 3.3.2 (http://www.R-project.org, The R Foundation) and Free Statistics software version 1.7.1. A two-tailed *p* value of < 0.05 was considered statistically significant.

## Results

### Baseline characteristics of subjects

From the MIMIC-IV database, we included adult patients who were diagnosed with AKI within 48 h of admission and were admitted to the ICU for the first time (N = 25,966). We excluded participants with less than three BG tests on the first day of ICU admission or had a stay of less than 24 h (N = 19,189). Consequently, this study ultimately resulting a total of 6777 patients for further analysis (Fig. 1). Overall, the average age of the participants was 63.5 ± 16.7 years, with 57.9% being male. Notably, the patients with high MBG levels (≥ 167.2 mg/dL) were found to have a lower hypoglycemia incidence, higher values for SBP, hemoglobin, platelets, WBC, Cr, and BUN. Additionally, these patients had a higher prevalence of myocardial infarct, congestive heart failure, cerebrovascular disease, kidney disease, sepsis, diabetes, and more frequent use of RRT, mechanical ventilation, vasoactive drugs and hypoglycemic drug compared to those in the other groups. Moreover, the patients exhibiting high CV levels (≥ 24.8%) were found to have lower values or incidences of hemoglobin, platelets, and hypertension, but higher values or incidences of WBC, Cr, BUN, myocardial infarct, liver disease, kidney disease, sepsis, diabetes, as well as increased use of RRT, vasoactive drugs and hypoglycemic drugs (Table 1).

**Table 1:** Baseline characteristics of participants.

Variables	All patients (n = 6777) Mean ± SD/Median (IQR)	MBG (mg/dL)				CV (%)			
		T1 < 125.0 (n = 2238)	125.0 ≤ T2 < 167.2 (n = 2280)	T3 ≥ 167.2 (n = 2259)	*p*-value	T1 < 12.7 (n = 2259)	12.7 ≤ T2 < 24.8 (n = 2259)	T3 ≥ 24.8 (n = 2259)	*p*-value
Age (years)	63.5 ± 16.7	61.5 ± 18.3	65.0 ± 16.7	64.0 ± 14.7	< 0.001	63.4 ± 17.2	63.6 ± 16.9	63.6 ± 16.0	0.940
Sex, Male, n (%)	3924 (57.9)	1275 (57.0)	1322 (58.0)	1327 (58.7)	0.482	1317 (58.3)	1296 (57.4)	1311 (58.0)	0.809
Vital signs									
Heart rate (bpm)	88.9 ± 17.9	89.0 ± 18.2	88.7 ± 17.5	89.0 ± 17.9	0.789	88.8 ± 17.8	88.5 ± 17.7	89.4 ± 18.1	0.187
SBP (mmHg)	115.4 ± 16.3	114.2 ± 16.2	115.7 ± 16.0	116.3 ± 16.7	< 0.001	115.6 ± 16.1	115.5 ± 16.3	115.1 ± 16.5	0.619
DBP (mmHg)	62.3 ± 11.0	62.2 ± 11.4	62.3 ± 10.9	62.3 ± 10.8	0.908	62.6 ± 11.1	62.4 ± 10.9	61.8 ± 11.0	0.068
SpO_2_ (%)	96.9 ± 2.5	96.8 ± 2.2	97.0 ± 2.5	96.8 ± 2.9	0.008	96.9 ± 2.2	96.9 ± 2.4	96.9 ± 2.9	0.863
Laboratory tests									
Hemoglobin (g/dL)	9.7 ± 2.3	9.5 ± 2.2	9.7 ± 2.4	9.8 ± 2.4	< 0.001	9.8 ± 2.3	9.8 ± 2.4	9.5 ± 2.3	< 0.001
Platelets (× 10^9^/L)	156.0 (100.0, 219.0)	151.0 (96.0, 212.0)	157.0 (102.0, 220.0)	161.0 (99.0, 224.0)	0.017	158.0 (104.0, 220.0)	158.0 (102.0, 219.0)	152.0 (89.0, 218.0)	0.006
WBC (× 10^9^/L)	14.6 (10.3, 20.4)	13.3 (9.4, 18.7)	14.6 (10.5, 20.1)	16.2 (11.4, 22.0)	< 0.001	13.9 (10.1, 19.1)	14.5 (10.2, 20.3)	15.6 (10.7, 21.7)	< 0.001
Na (mmol/L)	135.3 ± 6.6	134.8 ± 6.9	136.0 ± 6.0	135.0 ± 6.7	< 0.001	135.5 ± 6.3	135.4 ± 6.3	134.9 ± 7.1	0.004
Cr (mg/dl)	1.3 (0.9, 2.2)	1.3 (0.8, 2.4)	1.2 (0.8, 1.9)	1.4 (1.0, 2.2)	< 0.001	1.2 (0.8, 2.0)	1.3 (0.9, 2.1)	1.5 (1.0, 2.4)	< 0.001
BUN (mg/dl)	25.0 (16.0, 44.0)	25.0 (15.0, 44.0)	23.0 (15.0, 40.0)	28.0 (18.0, 47.0)	< 0.001	24.0 (15.0, 42.0)	24.0 (16.0, 43.0)	28.0 (18.0, 47.0)	< 0.001
Comorbidity diseases, n (%)									
Myocardial infarct	1404 (20.7)	349 (15.6)	431 (18.9)	624 (27.6)	< 0.001	430 (19.0)	458 (20.3)	516 (22.8)	0.006
Congestive heart failure	2302 (34.0)	721 (32.2)	753 (33.0)	828 (36.7)	0.004	785 (34.7)	761 (33.7)	756 (33.5)	0.622
Peripheral vascular disease	851 (12.6)	262 (11.7)	295 (12.9)	294 (13.0)	0.332	261 (11.6)	283 (12.5)	307 (13.6)	0.118
Cerebrovascular disease	818 (12.1)	246 (11.0)	254 (11.1)	318 (14.1)	0.002	285 (12.6)	256 (11.3)	277 (12.3)	0.392
Liver disease	1433 (21.1)	543 (24.3)	401 (17.6)	489 (21.6)	< 0.001	449 (19.9)	464 (20.5)	520 (23.0)	0.024
Kidney disease	1765 (26.0)	523 (23.4)	567 (24.9)	675 (29.9)	< 0.001	525 (23.2)	560 (24.8)	680 (30.1)	< 0.001
Malignant cancer	1039 (15.3)	343 (15.3)	344 (15.1)	352 (15.6)	0.899	336 (14.9)	358 (15.8)	345 (15.3)	0.659
Sepsis	4996 (73.7)	1595 (71.3)	1663 (72.9)	1738 (76.9)	< 0.001	1605 (71)	1643 (72.7)	1748 (77.4)	< 0.001
Diabetes	2307 (34.0)	340 (15.2)	603 (26.4)	1364 (60.4)	< 0.001	554 (24.5)	707 (31.3)	1046 (46.3)	< 0.001
Hypertension	3112 (45.9)	977 (43.7)	1099 (48.2)	1036 (45.9)	0.009	1075 (47.6)	1068 (47.3)	969 (42.9)	0.002
AKI stage, no. (%)					0.004				< 0.001
1	1666 (24.6)	576 (25.7)	547 (24.0)	543 (24.0)		538 (23.8)	575 (25.5)	553 (24.5)	
2	2844 (42.0)	927 (41.4)	1015 (44.5)	902 (39.9)		1036 (45.9)	960 (42.5)	848 (37.5)	
3	2267 (33.5)	735 (32.8)	718 (31.5)	814 (36.0)		685 (30.3)	724 (32.0)	858 (38.0)	
Interventions (day 1), n (%)									
RRT use	859 (12.7)	282 (12.6)	233 (10.2)	344 (15.2)	< 0.001	245 (10.8)	256 (11.3)	358 (15.8)	< 0.001
Mechanical ventilation	5071 (74.8)	1555 (69.5)	1753 (76.9)	1763 (78.0)	< 0.001	1662 (73.6)	1681 (74.4)	1728 (76.5)	0.066
Vasoactive drugs use	3863 (57.0)	1143 (51.1)	1301 (57.1)	1419 (62.8)	< 0.001	1166 (51.6)	1286 (56.9)	1411 (62.5)	< 0.001
Hypoglycemic drugs use	4790 (70.7)	1135 (50.7)	1584 (69.5)	2071 (91.7)	< 0.001	1401 (62.0)	1534 (67.9)	1855 (82.1)	< 0.001
Severity of illness									
SOFA score	4.0 (2.0, 5.0)	4.0 (2.0, 5.0)	3.0 (2.0, 5.0)	4.0 (2.0, 6.0)	< 0.001	3.0 (2.0, 5.0)	4.0 (2.0, 5.0)	4.0 (2.0, 6.0)	< 0.001
Comorbidity index	6.0 (4.0, 8.0)	6.0 (4.0, 8.0)	6.0 (4.0, 8.0)	7.0 (5.0, 9.0)	< 0.001	6.0 (4.0, 8.0)	6.0 (4.0, 8.0)	6.0 (4.0, 8.0)	< 0.001
Outcomes, n (%)									
ICU 30-day mortality	1593 (23.5)	497 (22.2)	478 (21.0)	618 (27.4)	< 0.001	468 (20.7)	495 (21.9)	630 (27.9)	< 0.001
Hospital mortality	1618 (23.9)	507 (22.7)	487 (21.4)	624 (27.6)	< 0.001	477 (21.1)	502 (22.2)	639 (28.3)	< 0.001
Hypoglycemia ara>	505 (7.5)	363 (16.2)	74 (3.2)	68 (3.0)	< 0.001	31 (1.4)	107 (4.7)	367 (16.2)	< 0.001

*MBG* mean blood glucose, *CV* coefficient of variation, *SBP* systolic blood pressure, *DBP* diastolic blood pressure, *SpO2* blood oxygen saturation, *WBC* white blood cell, *Na* sodium, *Cr* creatinine, *BUN* blood urea nitrogen, *RRT* renal replacement therapy, *SOFA* sequential organ failure assessment.

### Association between MBG, CV and ICU 30-day mortality in AKI

Table 1 shows that the ICU 30-day mortality rate for AKI patients was 23.5% (N = 1593). The results of univariate analyses of various covariates and their relation to mortality are provided in the Supplementary Information (Table S1). We constructed four multivariate Cox regression models to examine the independent effects of MBG and CV on ICU 30-day mortality. Hazard ratios (HRs) and 95% confidence intervals (CIs) are presented in Table 2. On a continuous scale, the multivariable Cox regression results showed that each SD increases in MBG and CV were associated with 1.20-fold (95% CI 1.14–1.27) and 1.08-fold (95% CI 1.03–1.13) increases in the risk of ICU 30-day mortality, respectively (Table 2, model 4). When AKI patients were divided into three tertiles according to their MBG and CV levels, it was found that compared with those with MBG levels < 125.0 mg/dl, AKI patients with MBG levels ≧167.2 mg/dl had a significantly increased risk of ICU 30-day mortality, with an HR of 1.34 (95% CI 1.17–1.53). While those with MBG levels ranging from 125.0 to 167.2 mg/dl did not exhibit a significant difference in risk, with an HR of 0.95 (95% CI 0.84–1.09). All the *P* values were < 0.05 in the trend tests (Table 2, model 4). Similarly, compared with those with CV levels < 12.7%, AKI patients with CV levels ≥ 24.8% had an increased risk of ICU 30-day mortality, with an HR of 1.37 (95% CI 1.21–1.56), while those with CV levels between 12.7 and 24.8% had no significant difference, with an HR of 1.05 (95% CI 0.92–1.18). All the *P* values were < 0.001 in the trend tests (Table 2, model 4).

**Table 2:** Multivariable-adjusted HRs and 95% CIs of MBG and CV associated with ICU 30-day mortality.

Variables	Unadjusted		Model 1		Model 2		Model 3		Model 4	
ICU 30-day mortality	HR (95%CI)	*p*-value	HR (95%CI)	*p*-value	HR (95%CI)	*p*-value	HR (95%CI)	*p*-value	HR (95%CI)	*p*-value
MBG (per one SD, mg/dl)	1.14 (1.09–1.19)	< 0.001	1.13 (1.08–1.19)	< 0.001	1.12 (1.07–1.18)	< 0.001	1.17 (1.11–1.23)	< 0.001	1.20 (1.14–1.27)	< 0.001
T1 (< 125.0)	Ref		Ref		Ref		Ref		Ref	
T2 (125.0–167.2)	0.89 (0.79–1.01)	0.071	0.84 (0.74–0.95)	0.006	0.87 (0.77–0.99)	0.035	0.92 (0.81–1.04)	0.177	0.95 (0.84–1.09)	0.475
T3 (≥ 167.2)	1.18 (1.05–1.33)	0.006	1.14 (1.01–1.28)	0.032	1.13 (1.00–1.27)	0.044	1.23 (1.08–1.40)	0.002	1.34 (1.17–1.53)	< 0.001
*P* for trend		0.004		0.018		0.027		0.002		< 0.001
CV (per one SD, %)	1.11 (1.06–1.16)	< 0.001	1.10 (1.05–1.14)	< 0.001	1.08 (1.03–1.13)	0.001	1.09 (1.04–1.14)	< 0.001	1.08 (1.03–1.13)	0.001
T1 (< 12.7)	Ref		Ref		Ref		Ref		Ref	
T2 (12.7–24.8) ara>	1.06 (0.94–1.20)	0.353	1.05 (0.92–1.19)	0.471	1.02 (0.90–1.15)	0.798	1.04 (0.92–1.18)	0.531	1.04 (0.92–1.18)	0.534
T3 (≥ 24.8)	1.40 (1.24–1.57)	< 0.001	1.40 (1.24–1.57)	< 0.001	1.31 (1.17–1.48)	< 0.001	1.37 (1.21–1.55)	< 0.001	1.37 (1.21–1.56)	< 0.001
*P* for trend		< 0.001		< 0.001		< 0.001		< 0.001		< 0.001

Model 1 adjusted for age and sex.

Model 2 adjusted for Model 1+ heart rate, SBP, DBP, SpO2, platelets, WBC, Cr, and BUN.

Model 3 adjusted for Model 1+ Model 2+ myocardial infarct, cerebrovascular disease, liver disease, kidney disease, malignant cancer, sepsis, hypertension, and diabetes.

Model 4 adjusted for Model 1+ Model 2+ Model 3+ AKI stage, RRT use, mechanical ventilation, vasoactive drug use, hypoglycemic drug use, SOFA score, and comorbidity index.

*MBG* mean blood glucose, *CV* coefficient of variation, *SBP* systolic blood pressure, *DBP* diastolic blood pressure, *SpO2* blood oxygen saturation, *WBC* white blood cell, *Cr* creatinine, *BUN* blood urea nitrogen, *RRT* renal replacement therapy, *SOFA* sequential organ failure assessment.

**Table 3:** The non-linear relationship between MBG and ICU 30-day mortality.

Threshold of driving pressure	ICU 30-day mortality	
	HR (95%CI)	*p*-value
MBG (mg/dl)		
< 111.3	0.989 (0.980, 0.998)	0.0169
≥ 111.3	1.003 (1.002, 1.004)	< 0.001
Likelihood Ratio test	–	< 0.001

Adjusted for age, sex, heart rate, SBP, DBP, SpO2, platelets, WBC, Cr, BUN, myocardial infarct, cerebrovascular disease, liver disease, kidney disease, malignant cancer, sepsis, hypertension, diabetes, AKI stage, RRT use, mechanical ventilation, vasoactive drug use, hypoglycemic drug use, SOFA score, and comorbidity index.

*MBG* mean blood glucose, *SBP* systolic blood pressure, *DBP* diastolic blood pressure, *SpO2* blood oxygen saturation, *WBC* white blood cell, *Cr* creatinine, *BUN* blood urea nitrogen, *RRT* renal replacement therapy, *SOFA* sequential organ failure assessment.

**Figure 2:**
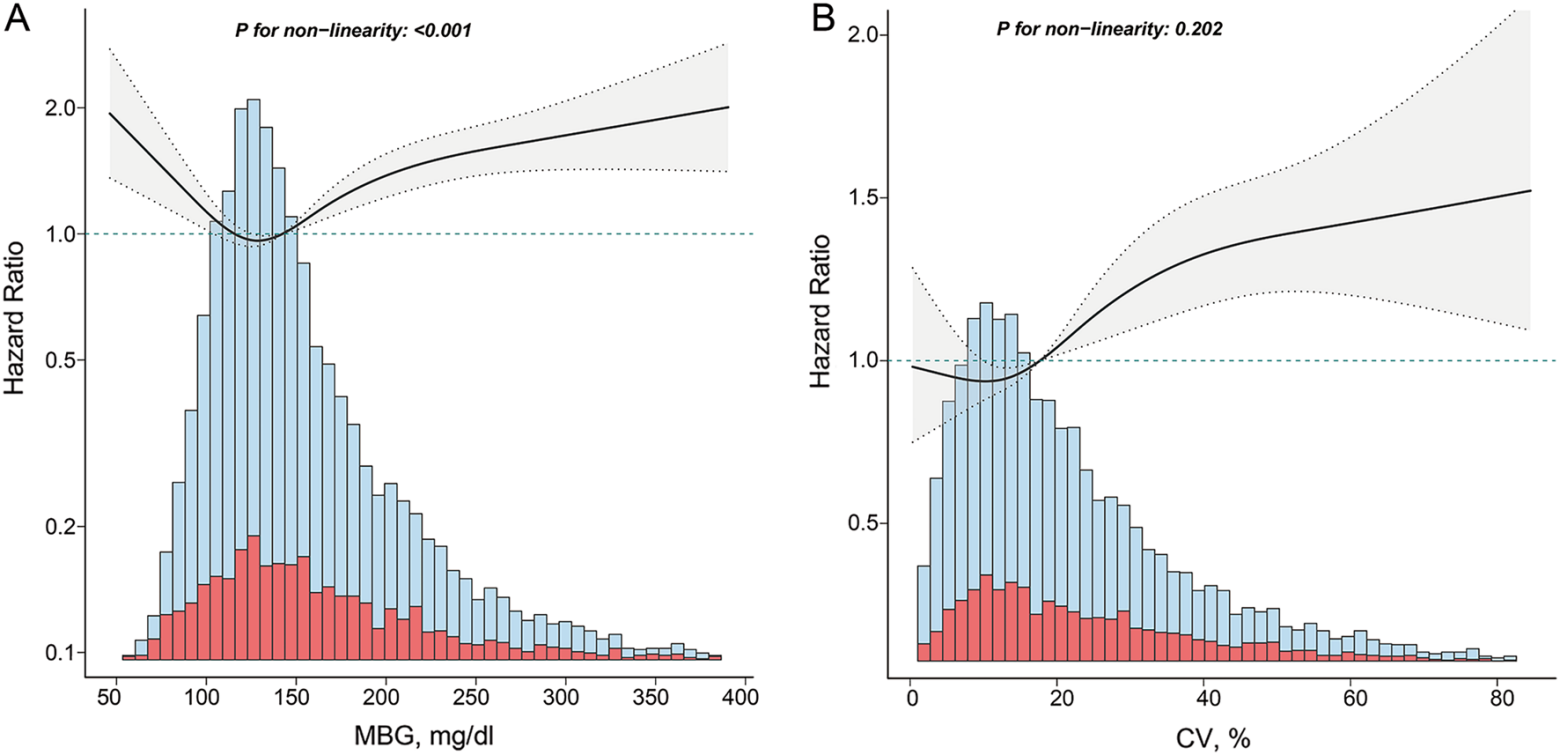
The relationship of MBG and CV associated with ICU 30-day mortality. (**A**) The non-linear relationship between MBG and ICU 30-day mortality. (**B**) The linear relationship between CV and ICU 30-day mortality. Hazard ratios (HRs) were adjusted for age, sex, heart rate, SBP, DBP, SpO2, platelets, WBC, Cr, BUN, myocardial infarct, cerebrovascular disease, liver disease, kidney disease, malignant cancer, sepsis, hypertension, diabetes, AKI stage, RRT use, mechanical ventilation, vasoactive drug use, hypoglycemic drug use, SOFA score, and comorbidity index. *MBG* mean blood glucose, *CV* coefficient of variation, *SBP* systolic blood pressure, *DBP* diastolic blood pressure, *SpO2* blood oxygen saturation, *WBC* white blood cell, *Cr* creatinine, *BUN* blood urea nitrogen, *RRT* renal replacement therapy, *SOFA* sequential organ failure assessment.

The smoothed curve fitting and restricted cubic spline analysis revealed that MBG levels had a U-shaped relationship with ICU 30-day mortality, with an inflection point for MBG at 111.3 mg/dl (Fig. 2A, Table 3). Utilizing segmented multivariate Cox regression models to accommodate the two distinct slopes around this inflection point, the analysis yielded the *P* value of < 0.001 for the likelihood ratio test (Table 3). On both sides of the inflection point, we found that the HR was 0.989 (95% CI 0.980–0.998) for MBG levels < 111.3 mg/dl; however, when MBG levels ≥ 111.3 mg/dl, the HR was 1.003 (95% CI 1.002–1.004) (Table 3). In contrast, the relationship between CV levels and ICU 30-day mortality was found to be linear, with an ascending trend in mortality risk as CV levels increased (Fig. 2B).

**Figure 3:**
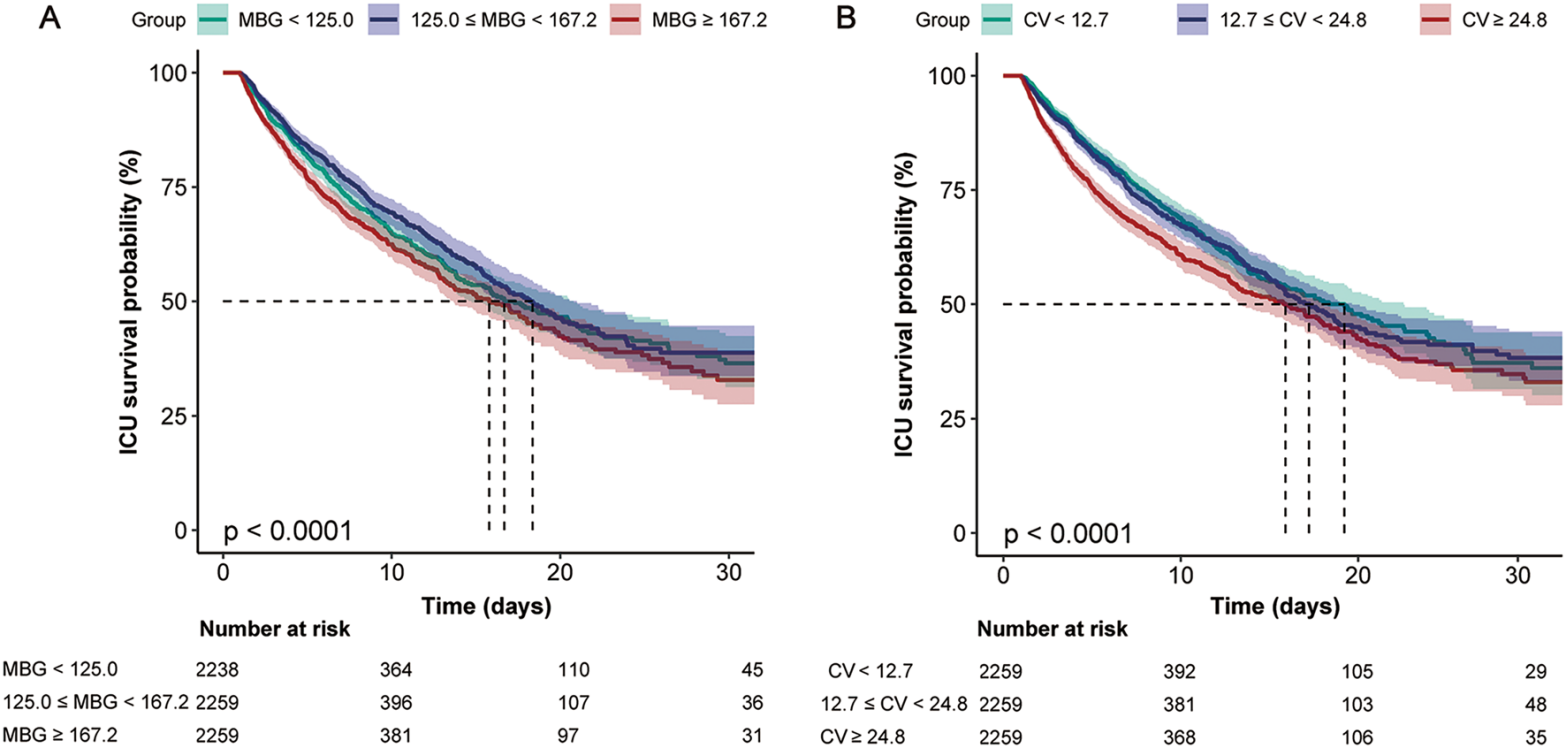
Kaplan–Meier survival curves for ICU 30-day mortality. (**A**) MBG (mg/dl) with ICU 30-day mortality; (**B**) CV (%) with ICU 30-day mortality. *MBG* mean blood glucose, *CV* coefficient of variation.

In addition, the Kaplan–Meier curves revealed that patients in the highest MBG tertile (T3) experienced the lowest ICU 30-day survival rates compared to other groups, which was similar to the findings in the highest CV tertile (T3) (Fig. 3). These results suggest that patients with higher MBG levels (≥ 167.2 mg/dl) and CV levels (≧ 24.8%) on the first day of ICU admission are associated with lower ICU 30-day survival (*P* < 0.0001).

### Sensitivity analysis

**Figure 4:**
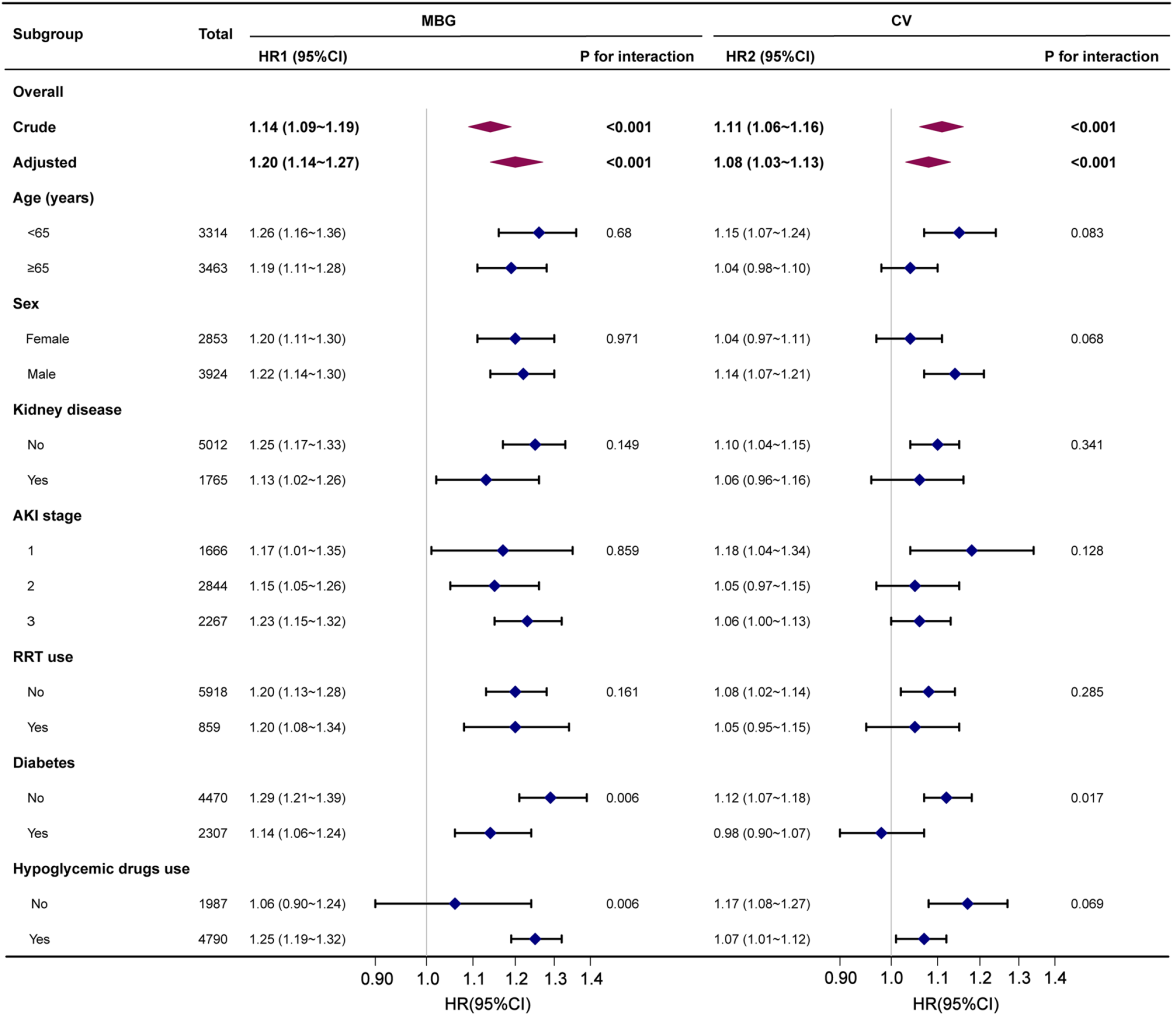
Subgroup analyses of the MBG and CV associated with ICU 30-day mortality. Hazard ratios (HRs) were adjusted for age, sex, heart rate, SBP, DBP, SpO2, platelets, WBC, Cr, BUN, myocardial infarct, cerebrovascular disease, liver disease, kidney disease, malignant cancer, sepsis, hypertension, diabetes, AKI stage, RRT use, mechanical ventilation, vasoactive drug use, hypoglycemic drug use, SOFA score, and comorbidity index. *MBG* mean blood glucose, *SBP* systolic blood pressure, *DBP* diastolic blood pressure, *SpO2* blood oxygen saturation, *WBC* white blood cell, *Cr* creatinine, *BUN* blood urea nitrogen, *RRT* renal replacement therapy, *SOFA* sequential organ failure assessment.

We used age (< 65, ≥ 65 years), sex (female, male), kidney disease, AKI stage, diabetes, use of RRT and hypoglycemic drugs as stratification variables to observe the effect values and generate a forest plot of data (Fig. 4). The interaction analysis indicated that MBG and CV were associated with a high risk of ICU 30-day mortality in patients with no diabetes. As the MBG and CV increased, the ICU 30-day mortality in the no-diabetes subgroup significantly increased (HR_MBG_: 1.29, 95% CI: 1.21–1.39, *P* = 0.006; HR_CV_: 1.12, 95% CI: 1.07–1.18, *P* = 0.017, respectively). The interaction between hypoglycemic drug use and the association of MBG with ICU 30-day mortality was also found to be significant (HR: 1.25, 95% CI: 1.19–1.32, *P* = 0.006) (Fig. 4).

In this study, the proportion of covariates with missing data was < 30%. The results of the Cox regression model applied to the original data exhibited consistent direction of effect values for the data before and after interpolation (Table S2). Following the exclusion of missing data from the full cohort (n = 6777), 4,948 patients were left, and the relationship between MBG, CV, and ICU 30-day mortality remained stable (Table S3) (Model 4: HR_MBG_: 1.19, 95% CI: 1.13–1.26, *P* < 0.001; HR_CV_: 1.07, 95% CI: 1.01–1.12, *P* = 0.011, respectively).

## Discussion

In this retrospective cohort study, we found that MBG and CV were independently associated with poor outcomes in ICU patients with AKI. There is a U-shaped relationship between the MBG levels and ICU 30-day mortality rate in AKI patients, with an inflection point of 111.3 mg/dl. When the MBG levels were < 111.3 mg/dl, a 1-mg/dl decrease in MBG was associated with a 1.1% reduction in the ICU 30-day mortality rate among AKI patients (HR 0.989, 95% CI 0.980–0.998, *P* = 0.0169). Conversely, when the MBG levels were ≥ 111.3 mg/dl, the ICU 30-day mortality rate increased by 0.3% for every 1-mg/dl increase in MBG (HR 1.003, 95% CI 1.002–1.004, *P* < 0.001). Moreover, we observed a linear relationship between the CV levels and ICU 30-day mortality, with the ICU 30-day mortality rate increasing by 11% for every 1-SD (17.86%) increase in CV (HR 1.11, 95% CI 1.06–1.16, *P* < 0.001). Elevated CV levels were significantly associated with lower survival rates in AKI patients. Consequently, maintaining BG levels within a reasonable range and minimizing glycemic variability represent effective strategies for mitigating the risk of mortality in AKI patients.

AKI has long been acknowledged as a complication of critical illness that is independently associated with mortality^23,24^. AKI is characterized by a variable etiology and complex pathogenesis. Previous studies have shown that infection, age, AKI stage, severity of circulatory shock, RRT treatment, comorbidities, and SOFA score are important factors that could affect the outcome of patients with AKI^23,25–27^. However, the evidence from studies on the relationship between BG levels and AKI outcomes remains limited. Bagshaw et al. analyzed the data of adult patients at 24 ICUs from 2000 to 2005 and found that both early hypoglycemia (BG < 4.5 mmol/L) and early variability in BG (< 4.5 and > or = 12.0 mmol/L) independently predicted an increased mortality risk^16^. Despite its large sample size, the study did not deeply explore the impact of AKI as a co-morbid condition on outcomes, and it defined BG variability solely based on the range of two randomly measured glucose values. The study by Gorelik et al. found that hyperglycemia (> 10 mmol/L) was positively associated with the incidence of AKI and the severity of acute illness, as well as an increase in 30-day mortality among non-diabetic inpatients^28^. A retrospective cohort study by Li et al. found that AKI patients should maintain their BG at a reasonable range, and should not drop lower than 5.52 mmol/L^10^. However, none of the aforementioned studies establish a conclusive link between various BG levels and mortality in AKI patients, and it is difficult to represent the overall BG levels during hospitalization based solely on the first BG values on admission. Therefore, the optimal BG control range still needs further study.

Recent studies have found that increased perioperative glucose variability is significantly associated with a heightened risk of postoperative AKI^29,30^. Consistently, a high glucose variability has been consistently associated with adverse prognosis in critically ill patients during their ICU stay^31,32^. Despite these findings, there remains a lack of consensus on the impact of glucose variability on mortality among AKI patients. In this study, we used all biochemical BG records from the first day of ICU admission to calculate the MBG and CV levels, thereby capturing the overall status of BG on the first day. Our results suggested that increased BMG and CV levels were associated with elevated mortality in AKI patients. Specifically, high BMG levels (≥ 167.2 mg/dl) and high CV levels (≥ 24.8%) were independently associated with an increased risk of ICU 30-day mortality among patients with AKI. In line with our findings, a secondary analysis from the Normal versus Augmented Level of RRT study showed that higher CV and SD values were associated with an increased risk of ICU 90-day death in severe AKI patients^33^. Unfortunately, this study focused on the relationship between baseline BG levels, glycemic variability and RRT-related outcomes in patients with AKI, which did not clearly elucidate other confounding factors that could influence clinical outcomes, such as diabetic status and the use of hypoglycemic drugs, nor did it establish a definitive BG control range.

There is inconsistency among guidelines regarding BG control goals for patients with AKI. The latest American Diabetes Association guidelines recommend a target glucose range of 7.8–10.0 mmol/L for patients with severe disease^34^. The KDIGO guidelines previously recommended a BG control target at 6.1–8.3 mmol/L for AKI patients^35^. In our study, the inflection point of the MBG levels in AKI patients was 111.3 mg/dl (6.18 mmol/L), indicating that these patients have the highest survival rate at this point. Notably, the inflection points in our study occurred in the T1 (< 125.0 mg/dl) group, while more patients had higher MBG levels, so only a weak protective effect was shown in the T2 (125.0–167.2 mg/dl) group, and the lowest survival rate was observed in the T3 (≥ 167.2 mg/dl) group due to higher MBG levels. We speculated that the reason for this phenomenon is that critically ill patients in the ICU often have elevated stress glucose, hence most patients have glucose values above the inflection point.

Glycemic variability represents a crucial aspect of glucose homeostasis, with its elevation signaling potential for extreme glycemic excursions, thereby increasing the risk for both hyperglycemia and hypoglycemia^36^. The CV is widely recognized as a key measure of glycemic variability^37,38^. In our study, we observed a higher mortality rate in the highest tertile of glycemic variability (T3, CV ≥ 24.8%), which suggested that reducing glycemic variability could improve survival outcomes in patients with AKI. This finding also confirmed the wide variability in glycemic control associated with significantly higher mortality, as suggested in previous studies^12,16,39^. Considering the potential effect of diabetic status on CV, we further performed multifactorial regression analysis by dividing the entire cohort into diabetic and non-diabetic groups. Our results showed that elevated glycemic variability is independently associated with increased risk of mortality among patients without diabetes mellitus, but this association was not observed in patients with diabetes mellitus (Table S4 and Table S5). This suggests that diabetic status may act as a protective effect against their adverse prognosis. We propose two possible explanations for this observation: (1) Patients with diabetes experience chronic hyperglycemia, which allows their bodies to adapt to fluctuations in BG levels and enhances their capacity to counteract stress-induced BG fluctuations^40,41^. However, non-diabetic patients with stress hyperglycemia might be less capable of regulating BG levels. This discrepancy can lead to acute oxidative stress production and release of large amounts of inflammatory factor^42,43^, which potentially cause pancreatic β-cell toxicity and irreversible impairment of pancreatic function^44^. (2) The increased risk of hypoglycemia due to elevated CV could mask the association between CV and mortality in AKI patients with diabetes. Further investigation needs to be done to uncover the underlying mechanisms responsible for this effect.

It is well known that the kidney, as an organ of gluconeogenesis, plays an important role in maintaining glucose metabolic homeostasis through glucose reabsorption and utilization^45^. The kidney is a mitochondria-rich organ^46^. In the high glucose state, mitochondria experience swelling and expansion, but cannot be cleared promptly. This malfunction leads to the release of large amounts of reactive oxygen species, contributing to the oxidative stress response in renal tubules. This oxidative stress plays a pivotal role in the progression of fibrosis and apoptosis of renal tubular epithelial cells, which in turn leads to reduced glucose reabsorption capacity of renal tubules, inducing renal fibrosis and failure^47,48^. Meanwhile, mitochondrial damage, characterized by fragmentation and the release of damage-associated molecular patterns has been shown to induce and accelerate the development of AKI^49^. In addition, hyperglycemia exacerbates renal ischemia–reperfusion injury/hypoxia-reperfusion injury (HRI) induced AKI^50^. This condition has been strongly associated with all-cause mortality in patients with critical illness^51–53^. Consistently, this conclusion was also confirmed in in vitro experiments^54^. The primary pathophysiological mechanism is closely associated with severe hyperglycemia leading to dehydration, reduced circulating blood volume, acidosis, infection, and ultimately resulting in rhabdomyolysis and multiple organ dysfunction^55,56^. Hypoglycemia is comparable to diabetic ketoacidosis and hyperosmolar nonketotic diabetic coma, with acute cardiovascular and cerebrovascular disease due to insufficient energy supply to the heart and brain cells as the main mortality event^57^. There is insufficient evidence regarding the direct cause of kidney injury by hypoglycemia, and the possible mechanisms need to be further explored.

Our study offers several notable advantages. First, this is a real-world study with a large sample, which is highly generalizable. Second, by utilizing the MBG and CV on the first day of admission as variables to study the effect on the outcome, we mitigated the risk of false inferences on outcome caused by one random BG fluctuation—thereby enhancing the stability of our findings. Third, we employed rigorous statistical methods to minimize the impact of residual confounders on our findings. Finally, to the best of our knowledge, this is the first study demonstrating an independent association between glycemic variability and short-term mortality in ICU patients with AKI. The results of this study will help to improve the prognosis of AKI patients by informing more precise glycemia management strategies.

However, there are some limitations to our study that should be considered. First, due to the inherent nature of retrospective cohort studies, multiple confounding factors are inevitable. Thus, we conducted a multifactorial analysis to adjust for the effect of confounding factors on the study outcome as much as possible. Second, as an observational study, causality cannot be fully deduced. Third, for patients with multiple ICU admissions, we only analyzed baseline information from the first admission, which may have created a selection bias. Fourth, the inclusion of AKI patients was incomplete due to less than three BG tests on the first day of admission, which may have influenced the study's outcomes. Finally, although high CV was related to the presence of diabetes mellitus, the lack of detailed information about insulin dosage, duration of diabetes, and other possible diabetes-related complications, which means that confounding related to diabetes mellitus may not have been fully addressed.

## Conclusion

MBG and CV were significantly associated with short-term mortality in intensive care patients with AKI. Tighter glycemic control may be an effective measure to improve the prognosis of AKI patients.

### Supplementary Information

Supplementary Information.

## Data Availability

The raw data supporting the conclusions of this article will be made available by the the first author (Email: gyf0726@126.com), without undue reservation.
