# Supplementary material for: Association between glycemic variability and short-term mortality in patients with acute kidney injury: a retrospective cohort study of the MIMIC-IV database
Source: Sci Rep. 2024 Mar 11;14:5945. doi: 10.1038/s41598-024-56564-7 (PMC10928232; doi:10.1038/s41598-024-56564-7)
Supplement: Supplementary file 1 — Supplementary Information. [file 41598_2024_56564_MOESM1_ESM.docx]

**Association Between Glycemic Variability and Short-term Mortality in Patients With Acute Kidney Injury: A Retrospective Cohort Study of the MIMIC-IV Database**

Yifan Guo^1,2†^, Yue Qiu^3†^, Taiqi Xue^1^, Yi Zhou^1,2^, Pu Yan^1^, Shiyi Liu^1^, Shiwei Liu^1^, Wenjing Zhao^4*^, Ning Zhang^1*^

^1^Department of Nephropathy, Wang Jing Hospital of China Academy of Chinese Medical Sciences, Beijing, China

^2^Graduate School, Beijing University of Chinese Medicine, Beijing, China

^3^Beijing Miyun Hospital of Traditional Chinese Medicine, Miyun area, Beijing, China

^4^Department of Nephrology, Beijing Hospital of Traditional Chinese Medicine, Capital Medical University, Beijing, China

^†^These authors contributed equally to this work.

^*^Correspondence: [znice3927@126.com;](mailto:znice3927@126.com;) zhaowenjing@bjzhongyi.com.

**Table S1 Results of univariate analysis of ICU 30-day mortality.**

| **Variables** | **ICU 30-day mortality** | |
| --- | --- | --- |
|  | **HR (95%CI)** | ***p*-value** |
| Age (years)✝ | 1.37 (1.30,1.45) | < 0.001 |
| Sex (female vs. male ) | 0.92 (0.83,1.01) | 0.082 |
| **Vital Signs** |  |  |
| Heart rate (bpm)✝ | 1.00 (0.95,1.05) | 0.986 |
| SBP (mmHg)✝ | 0.80 (0.76,0.85) | < 0.001 |
| DBP (mmHg)✝ | 0.85 (0.80,0.90) | < 0.001 |
| SpO2 (%)✝ | 0.86 (0.83,0.90) | < 0.001 |
| **Laboratory tests** |  |  |
| Hemoglobin (g/L)✝ | 0.90 (0.86,0.95) | < 0.001 |
| Platelets (10^9/L)✝ | 0.90 (0.85,0.95) | < 0.001 |
| WBC (10^9/L)✝ | 1.07 (1.04,1.10) | < 0.001 |
| Na (mmol/L)✝ | 0.99 (0.94,1.04) | 0.557 |
| Cr (mg/dl)✝ | 1.08 (1.04,1.14) | < 0.001 |
| BUN (mg/dl)✝ | 1.20 (1.15,1.25) | < 0.001 |
| **Comorbidity disease, n (%)** |  |  |
| Myocardial infarct↗ | 1.36 (1.22,1.53) | < 0.001 |
| Congestive heart failure↗ | 1.04 (0.94,1.15) | 0.467 |
| Peripheral vascular disease↗ | 1.05 (0.91,1.21) | 0.495 |
| Cerebrovascular disease↗ | 1.16 (1.02,1.32) | 0.027 |
| Liver disease↗ | 1.36 (1.22,1.52) | < 0.001 |
| Kidney disease↗ | 1.15 (1.03,1.29) | 0.012 |
| Malignant cancer↗ | 1.45 (1.27,1.65) | < 0.001 |
| Sepsis↗ | 0.80 (0.69,0.92) | 0.003 |
| Hypertension↗ | 1.01 (0.92,1.11) | 0.847 |
| Diabetes↗ | 0.92 (0.82,1.02) | 0.104 |
| **AKI stage, no. (%)** |  |  |
| 1 | ref |  |
| 2 | 1.14 (0.97,1.32) | 0.106 |
| 3 | 1.79 (1.55,2.07) | < 0.001 |
| **Interventions (day 1), n (%)** |  |  |
| RRT use | 1.08 (0.96,1.21) | 0.223 |
| Mechanical ventilation | 1.07 (0.90,1.26) | 0.438 |
| Vasoactive drug use | 1.58 (1.39,1.79) | < 0.001 |
| Hypoglycemic drug use | 0.68 (0.61,0.76) | < 0.001 |
| **Severity of illness** |  |  |
| SOFA score | 1.06 (1.05,1.08) | < 0.001 |
| Comorbidity index | 1.10 (1.08,1.12) | < 0.001 |
| **Blood glucose related (day 1)** |  |  |
| MBG (mg/dl)✝ | 1.14 (1.09,1.19) | < 0.001 |
| CV (%)✝ | 1.14 (1.10,1.18) | < 0.001 |
| Hypoglycemia (%)↗ | 1.70 (1.46,1.97) | < 0.001 |

✝: The continuous variables goes up by one SD.

↗: The categorical variables are referenced by ‘No’.

MBG, mean blood glucose; CV, coefficient of variation; SBP, systolic blood pressure; DBP, diastolic blood pressure; SpO2, blood oxygen saturation; WBC, white blood cell; Na, sodium; Cr, creatinine; BUN, blood urea nitrogen; RRT, renal replacement therapy; SOFA, sequential organ failure assessment.

**Table S2 Multivariable-adjusted HRs and 95% CIs of MBG and CV associated with ICU 30-day mortality (Before multiple imputations).**

| **Variables** | **Unadjusted** |  | **Model 1** | | **Model 2** | | **Model 3** | | **Model 4** | |
| --- | --- | --- | --- | --- | --- | --- | --- | --- | --- | --- |
| **ICU 30-day mortality** | **HR (95%CI)** | ***p*-value** | **HR (95%CI)** | ***p*-value** | **HR (95%CI)** | ***p*-value** | **HR (95%CI)** | ***p*-value** | **HR (95%CI)** | ***p*-value** |
| MBG (per one SD, mg/dl) | 1.14 (1.09~1.19) | <0.001 | 1.13 (1.08~1.19) | <0.001 | 1.12 (1.07~1.18) | <0.001 | 1.17 (1.11~1.23) | <0.001 | 1.19 (1.12~1.25) | <0.001 |
| T1 (< 125.0) | ref |  | ref |  | ref |  | ref |  | ref |  |
| T2 (125.0~167.2) | 0.89 (0.79~1.01) | 0.071 | 0.84 (0.74~0.95) | 0.006 | 0.88 (0.77~1.00) | 0.042 | 0.92 (0.81~1.05) | 0.215 | 0.92 (0.80~1.06) | 0.275 |
| T3 (≥167.2) | 1.18 (1.05~1.33) | 0.006 | 1.14 (1.01~1.28) | 0.032 | 1.13 (1.01~1.28) | 0.039 | 1.23 (1.08~1.41) | 0.001 | 1.28 (1.11~1.48) | 0.001 |
| *P* for trend |  | 0.004 |  | 0.018 |  | 0.025 |  | 0.001 |  | 0.001 |
| CV (per one SD, %) | 1.11 (1.06~1.16) | <0.001 | 1.10 (1.05~1.14) | <0.001 | 1.08 (1.03~1.13) | 0.001 | 1.09 (1.04~1.14) | <0.001 | 1.06 (1.01~1.12) | 0.011 |
| T1 (<12.7) | ref |  | ref |  | ref |  | ref |  | ref |  |
| T2 (12.7~24.8) | 1.06 (0.94~1.20) | 0.353 | 1.05 (0.92~1.19) | 0.471 | 1.01 (0.89~1.15) | 0.840 | 1.04 (0.91~1.18) | 0.572 | 1.03 (0.89~1.18) | 0.710 |
| T3 (≥24.8) | 1.40 (1.24~1.57) | <0.001 | 1.40 (1.24~1.57) | <0.001 | 1.31 (1.16~1.48) | <0.001 | 1.36 (1.21~1.54) | <0.001 | 1.33 (1.16~1.52) | <0.001 |
| *P* for trend |  | <0.001 |  | <0.001 |  | <0.001 |  | <0.001 |  | <0.001 |

Model 1 adjusted for age and sex.

Model 2 adjusted for Model 1+heart rate, SBP, DBP, SpO2, platelets, WBC, Cr, and BUN.

Model 3 adjusted for Model 1+Model 2+myocardial infarct, cerebrovascular disease, liver disease, kidney disease, malignant cancer, sepsis, hypertension, and diabetes.

Model 4 adjusted for Model 1+Model 2+Model 3+AKI stage, RRT use, mechanical ventilation, vasoactive drug use, hypoglycemic drug use, SOFA score, and comorbidity index.

MBG, mean blood glucose; CV, coefficient of variation; SBP, systolic blood pressure; DBP, diastolic blood pressure; SpO2, blood oxygen saturation; WBC, white blood cell; Cr, creatinine; BUN, blood urea nitrogen; RRT, renal replacement therapy; SOFA, sequential organ failure assessment.

**Table S3 Multivariable-adjusted HRs and 95% CIs of MBG and CV associated with ICU 30-day mortality (Exclude all missing data).**

| **Variables** | **Unadjusted** |  | **Model 1** | | **Model 2** | | **Model 3** | | **Model 4** | |
| --- | --- | --- | --- | --- | --- | --- | --- | --- | --- | --- |
| **ICU 30-day mortality** | **HR (95%CI)** | ***p*-value** | **HR (95%CI)** | ***p*-value** | **HR (95%CI)** | ***p*-value** | **HR (95%CI)** | ***p*-value** | **HR (95%CI)** | ***p*-value** |
| MBG (per one SD, mg/dl) | 1.13 (1.07~1.19) | <0.001 | 1.13 (1.07~1.19) | <0.001 | 1.12 (1.06~1.18) | <0.001 | 1.15 (1.09~1.22) | <0.001 | 1.19 (1.13~1.26) | <0.001 |
| T1 (< 126.4) | ref |  | ref |  | ref |  | ref |  | ref |  |
| T2 (126.4~170.0) | 0.85 (0.74~0.97) | 0.017 | 0.80 (0.70~0.91) | 0.001 | 0.84 (0.73~0.96) | 0.011 | 0.88 (0.76~1.00) | 0.058 | 0.91 (0.79~1.05) | 0.199 |
| T3 (≥170.0) | 1.13 (0.99~1.28) | 0.068 | 1.09 (0.96~1.24) | 0.193 | 1.09 (0.96~1.24) | 0.193 | 1.14 (1.00~1.31) | 0.056 | 1.27 (1.1~1.47) | 0.001 |
| *P* for trend |  | 0.058 |  | 0.156 |  | 0.160 |  | 0.063 |  | 0.002 |
| CV (per one SD, %) | 1.10 (1.05~1.16) | <0.001 | 1.09 (1.04~1.15) | <0.001 | 1.08 (1.02~1.13) | 0.003 | 1.08 (1.02~1.13) | 0.003 | 1.07 (1.01~1.12) | 0.011 |
| T1 (<13.0) | ref |  | ref |  | ref |  | ref |  | ref |  |
| T2 (13.0~25.6) | 1.07 (0.94~1.23) | 0.314 | 1.06 (0.92~1.21) | 0.424 | 1.01 (0.88~1.15) | 0.927 | 1.02 (0.89~1.17) | 0.750 | 1.02 (0.89~1.17) | 0.741 |
| T3 (≥25.6) | 1.39 (1.22~1.58) | <0.001 | 1.39 (1.22~1.59) | <0.001 | 1.30 (1.14~1.48) | <0.001 | 1.32 (1.16~1.51) | <0.001 | 1.33 (1.16~1.52) | <0.001 |
| *P* for trend |  | <0.001 |  | <0.001 |  | <0.001 |  | <0.001 |  | <0.001 |

Model 1 adjusted for age and sex.

Model 2 adjusted for Model 1+heart rate, SBP, DBP, SpO2, platelets, WBC, Cr, and BUN.

Model 3 adjusted for Model 1+Model 2+myocardial infarct, cerebrovascular disease, liver disease, kidney disease, malignant cancer, sepsis, hypertension, and diabetes.

Model 4 adjusted for Model 1+Model 2+Model 3+AKI stage, RRT use, mechanical ventilation, vasoactive drug use, hypoglycemic drug use, SOFA score, and comorbidity index.

MBG, mean blood glucose; CV, coefficient of variation; SBP, systolic blood pressure; DBP, diastolic blood pressure; SpO2, blood oxygen saturation; WBC, white blood cell; Cr, creatinine; BUN, blood urea nitrogen; RRT, renal replacement therapy; SOFA, sequential organ failure assessment.

**Table S4 Multivariable-adjusted HRs and 95% CIs of CV associated with ICU 30-day mortality in diabetic patients.**

| **Variables** | **Unadjusted** |  | **Model 1** | | **Model 2** | | **Model 3** | | **Model 4** | |
| --- | --- | --- | --- | --- | --- | --- | --- | --- | --- | --- |
| **ICU 30-day mortality** | **HR (95%CI)** | ***p*-value** | **HR (95%CI)** | ***p*-value** | **HR (95%CI)** | ***p*-value** | **HR (95%CI)** | ***p*-value** | **HR (95%CI)** | ***p*-value** |
| CV (per one SD, %) | 0.99 (0.90~1.08) | 0.821 | 0.99 (0.91~1.09) | 0.862 | 0.99 (0.90~1.09) | 0.847 | 0.99 (0.90~1.09) | 0.834 | 0.98 (0.89~1.07) | 0.666 |
| T1 (<12.7) | ref |  | ref |  | ref |  | ref |  | ref |  |
| T2 (12.7~24.8) | 0.97 (0.76~1.23) | 0.781 | 0.94 (0.74~1.19) | 0.601 | 0.92 (0.72~1.17) | 0.492 | 0.92 (0.72~1.17) | 0.500 | 0.91 (0.71~1.16) | 0.426 |
| T3 (≥24.8) | 1.05 (0.84~1.31) | 0.651 | 1.06 (0.85~1.32) | 0.578 | 1.03 (0.83~1.28) | 0.796 | 1.04 (0.83~1.29) | 0.747 | 1.02 (0.82~1.28) | 0.833 |
| *P* for trend |  | 0.585 |  | 0.482 |  | 0.683 |  | 0.633 |  | 0.698 |

Model 1 adjusted for age and sex.

Model 2 adjusted for Model 1+heart rate, SBP, DBP, SpO2, platelets, WBC, Cr, and BUN.

Model 3 adjusted for Model 1+Model 2+myocardial infarct, cerebrovascular disease, liver disease, kidney disease, malignant cancer, sepsis, hypertension.

Model 4 adjusted for Model 1+Model 2+Model 3+AKI stage, RRT use, mechanical ventilation, vasoactive drug use, hypoglycemic drug use, SOFA score, and comorbidity index.

MBG, mean blood glucose; CV, coefficient of variation; SBP, systolic blood pressure; DBP, diastolic blood pressure; SpO2, blood oxygen saturation; WBC, white blood cell; Cr, creatinine; BUN, blood urea nitrogen; RRT, renal replacement therapy; SOFA, sequential organ failure assessment.

**Table S5 Multivariable-adjusted HRs and 95% CIs of CV associated with ICU 30-day mortality in non-diabetic patients.**

| **Variables** | **Unadjusted** |  | **Model 1** | | **Model 2** | | **Model 3** | | **Model 4** | |
| --- | --- | --- | --- | --- | --- | --- | --- | --- | --- | --- |
| **ICU 30-day mortality** | **HR (95%CI)** | ***p*-value** | **HR (95%CI)** | ***p*-value** | **HR (95%CI)** | ***p*-value** | **HR (95%CI)** | ***p*-value** | **HR (95%CI)** | ***p*-value** |
| CV (per one SD, %) | 1.16 (1.11~1.22) | <0.001 | 1.15 (1.10~1.20) | <0.001 | 1.12 (1.07~1.18) | <0.001 | 1.13 (1.08~1.18) | <0.001 | 1.11 (1.06~1.17) | <0.001 |
| T1 (<12.7) | ref |  | ref |  | ref |  | ref |  | ref |  |
| T2 (12.7~24.8) | 1.10 (0.95~1.28) | 0.203 | 1.09 (0.94~1.27) | 0.236 | 1.06 (0.92~1.23) | 0.416 | 1.08 (0.93~1.26) | 0.307 | 1.08 (0.93~1.26) | 0.285 |
| T3 (≥24.8) | 1.65 (1.43~1.91) | <0.001 | 1.67 (1.44~1.93) | <0.001 | 1.54 (1.33~1.78) | <0.001 | 1.55 (1.34~1.79) | <0.001 | 1.56 (1.34~1.81) | <0.001 |
| *P* for trend |  | <0.001 |  | <0.001 |  | <0.001 |  | <0.001 |  | <0.001 |

Model 1 adjusted for age and sex.

Model 2 adjusted for Model 1+heart rate, SBP, DBP, SpO2, platelets, WBC, Cr, and BUN.

Model 3 adjusted for Model 1+Model 2+myocardial infarct, cerebrovascular disease, liver disease, kidney disease, malignant cancer, sepsis, hypertension.

Model 4 adjusted for Model 1+Model 2+Model 3+AKI stage, RRT use, mechanical ventilation, vasoactive drug use, hypoglycemic drug use, SOFA score, and comorbidity index.

MBG, mean blood glucose; CV, coefficient of variation; SBP, systolic blood pressure; DBP, diastolic blood pressure; SpO2, blood oxygen saturation; WBC, white blood cell; Cr, creatinine; BUN, blood urea nitrogen; RRT, renal replacement therapy; SOFA, sequential organ failure assessment.
